# Relationship between Plasma Triglyceride Level and Severity of Hypertriglyceridemic Pancreatitis

**DOI:** 10.1371/journal.pone.0163984

**Published:** 2016-10-11

**Authors:** Sheng-Huei Wang, Yu-Ching Chou, Wei-Chuan Shangkuan, Kuang-Yu Wei, Yu-Han Pan, Hung-Che Lin

**Affiliations:** 1 Division of Pulmonary and Critical Care Medicine, Department of Internal Medicine, Tri-Service General Hospital, National Defense Medical Center, Taipei, Taiwan, Republic of China; 2 School of Public Health, National Defense Medical Center, Taipei, Taiwan, Republic of China; 3 National Defense Medical Center, Taipei, Taiwan, Republic of China; 4 Division of Nephrology, Department of Internal Medicine, Tri-Service General Hospital, National Defense Medical Center, Taipei, Taiwan, Republic of China; 5 Department of Nursing, Tri-Service General Hospital, National Defense Medical Center, Taipei, Taiwan, Republic of China; 6 Department of Otolaryngology-Head and Neck Surgery, Tri-Service General Hospital, National Defense Medical Center, Taipei, Taiwan, Republic of China; University of Szeged, HUNGARY

## Abstract

**Background:**

Hypertriglyceridemia is the third most common cause of acute pancreatitis, but whether the level of triglyceride (TG) is related to severity of pancreatitis is unclear.

**Aim:**

To evaluate the effect of TG level on the severity of hypertriglyceridemic pancreatitis (HTGP).

**Design:**

Retrospective cohort study.

**Methods:**

We reviewed the records of 144 patients with HTGP from 1999 to 2013 at Tri-Service General Hospital. Patients with possible etiology of pancreatitis, such as gallstones, those consuming alcohol or drugs, or those with infections were excluded. The classification of severity of pancreatitis was based on the revised Atlanta classification. We allocated the patients into high-TG and low-TG groups based on the optimal cut-off value (2648 mg/dL), which was derived from the receiver operating characteristic (ROC) curve between TG level and severity of HTGP. We then compared the clinical characteristics, pancreatitis severity, and mortality rates of the groups.

**Results:**

There were 66 patients in the low-TG group and 78 patients in the high-TG group. There was no significant difference in the age, sex ratio, body mass index, and comorbidity between the 2 groups. The high-TG group had significantly higher levels of glucose (*P* = 0.022), total cholesterol (*P* = 0.002), and blood urea nitrogen (*P* = 0.037), and lower levels of sodium (*P* = 0.003) and bicarbonate (*P* = 0.002) than the low-TG group. The incidences of local complication (*P* = 0.002) and severe and moderate form of pancreatitis (*P* = 0.004) were significantly higher in the high-TG group than in the low-TG group. The mortality rate was higher in the high-TG group than in the low-TG group (*P* = 0.07).

**Conclusions:**

Higher TG level in patients with HTGP may be associated with adverse prognosis, but randomized and prospective studies are needed in the future verify this relationship.

## Introduction

Hypertriglyceridemia (HTG) is the third most common cause of acute pancreatitis (AP) after alcohol consumption and gallstones and accounts for 1–7% of patients with AP [1–5]. The etiology of gestational pancreatitis is considered to be attributed to HTG in approximately 56% of patients [6]. The percentage of cases in which hypertriglyceridemic pancreatitis (HTGP) causes AP is increasing worldwide [7–8]. The mechanism of HTG that causes HTGP is unclear, but some hypotheses have been proposed. The most accepted theory involves accumulation of free fatty acids in the pancreas, which results from excess triglyceride (TG) being hydrolyzed by pancreatic lipase. The free fatty acids may cause acinar cell and pancreatic capillary injury. Ischemia can create an acidic environment, which further reinforces the toxicity of the free fatty acids [9–10]. Another theory is hyperviscosity in pancreatic capillaries caused by high-level chylomicrons that could lead to ischemia and acidosis in the pancreas. Furthermore, endoplasmic reticulum stress is involved in the pathogenesis of HTGP [11]. The clinical presentation of HTGP (including abdominal pain, nausea, and vomiting) is similar to that of any other etiology, which is exacerbated with eating. In addition to conventional management of AP, such as aggressive intravenous hydration, analgesia, and fasting, patients with HTGP may experience benefits from plasma exchange, insulin, or heparin [12–14].

HTGP appears to have a disease course more severe than that of AP with other etiologies [15–17]. The risk of AP is approximately 5% in patients with serum TG level > 1000 mg/dL and approximately 10–20% in patients with TG level > 2000 mg/dL [18]. Serum TG levels of approximately ≥ 1000 mg/dL are indicative of HTG being the etiology of AP [19–20]. Hypertriglyceridemia should be highly suspected as the cause of AP in patients with serum TG levels between 500 and 1000 mg/dL if no other etiology of AP is present [21]. However, there have been conflicting results about the association of TG level and severity of HTGP [16, 21–24]. In this retrospective study, we reviewed the records of patients with HTGP, allocated them to the high-TG and low-TG groups according to the optimal cut-off value (2648 mg/dL), which was derived from the receiver operating characteristic (ROC) curve between TG level and severity of HTGP, and then compared the severity and mortality rates between these groups.

## Methods

### Patients and Data Collection

A total of 144 patients clinically diagnosed as having HTGP from January 1, 1999 to December 31, 2013 at the Tri-service General Hospital, National Defense Medical Center, Taiwan, were included in this retrospective study. The diagnosis of HTGP met all of the following criteria: (1) diagnosis of AP (requiring 2 of the following 3: acute onset epigastric pain often radiating to the back, elevation of amylase or lipase level to ≥ 3 times higher than the upper normal limit, and evidence of AP on abdominal computed tomography) and (2) serum TG level ≥ 1000 mg/dL. Exclusion criteria were: (1) an etiology of AP in addition to HTG, such as gallstones, alcohol and drug consumption, and infection and (2) history of AP. The clinical characteristics of the patients including age, sex, height, weight, body mass index, comorbidities such as diabetes, hypertension, or coronary artery disease, and levels of lipase, amylase, sodium, glucose, albumin, total cholesterol, lactate dehydrogenase (LDH), uric acid, C-reactive protein (CRP), blood urea nitrogen (BUN), and creatinine were analyzed. The biochemical and abdominal computed tomography data we collected had been obtained at initial presentation or within 2 days of admission. Then, the receiver operating characteristic (ROC) curve between TG level and severity of pancreatitis was used to determine the optimal cut-off value for patient allocation. The patients were allocated into 2 groups (high-TG group and low-TG group) on the basis of this value (2648 mg/dL). Specifically, the low-TG group included patients with TG levels between 1000 and 2648 mg/dL, and the high-TG group included patients with TG levels > 2648 mg/dL. This study was approved by the Institutional Review Board of Tri-Service General Hospital.

### Outcome Measures and Definitions

The primary outcomes were the severity of HTGP in the high-TG and low-TG groups, as evaluated according to the revised Atlanta classification [25]. Mild AP was defined as the absence of organ failure and local or systemic complications. Moderately severe AP was defined as the presence of transient organ failure, local complications, or systemic complications, with no persistent organ failure. Severe AP was defined as the presence of persistent organ failure. Organ failure was defined according to the modified Marshall scoring system (Table 1). Transient organ failure was defined as organ failure persisting < 48 h. Persistent organ failure was defined as organ failure continuing > 48 h. Local complications included acute necrotic collection (ANC), acute peripancreatic fluid collection (APFC), pancreatic pseudocyst, and walled-off necrosis. Systemic complication was defined as exacerbation of a pre-existing comorbidity precipitated by AP.

**Table 1:** Modified Marshall Scoring System for Organ Dysfunction.

Organ system	Score: 0	1	2	3	4
**Respiratory (PaO**_**2**_**/FiO**_**2**_**)**	>400	301–400	201–300	101–200	≤101
**Renal (serum creatinine, mg/dl)**	<1.4	1.4–1.8	1.9–3.6	3.6–4.9	>4.9
**Cardiovascular (systolic blood pressure, mm Hg)** #	>90	<90, fluid response	<90, no fluid response	<90, pH <7.3	<90, pH <7.2

A score of ≥2 in any system defines the presence of organ failure.

Organ failure resolving ≤48 h is defined as transient organ failure; organ failure persisting >48 h is defined as persistent organ failure

# without inotropic agents

Reference: 25

The secondary outcomes were intensive care unit (ICU) admission, hospitalization length, mortality, and recurrence of HTGP in 1 year after discharge. All patients were treated with conventional management and did not receive plasma exchange, insulin, or heparin therapy for HTGP. Insulin was administered only for patients who had diabetes with poor control.

### Statistical Analysis

Categorical variables were expressed as percentages, and continuous variables were expressed as mean ± standard deviation. The differences in categorical and continuous variables were analyzed by using the independent t-test, chi-square test, or Fisher’s exact test. A *P* < 0.05 was considered statistically significant. Statistical evaluation was performed by using SPSS software version 18.0 (SPSS Inc., Chicago, IL).

## Results

### Study Population Characteristics

**Fig 1:**
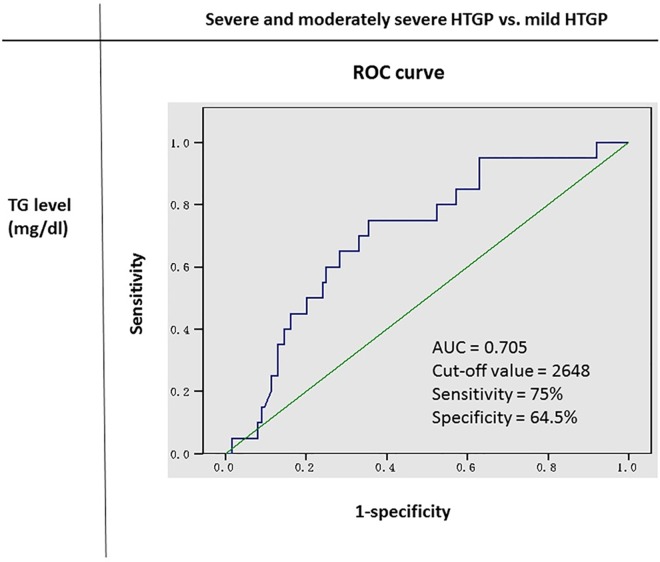
ROC Analysis of TG Level to Pancreatitis Severity among HTGP Patients.

This study included 144 patients with HTGP hospitalized from 1999 to 2013. The ROC curve showed that a TG level of 2648 mg/dL was the optimal cut-off value for predicting more severe form of HTGP. The area under the ROC curve was 0.705. The sensitivity and specificity was 75% and 64.5%, respectively (Fig 1). All patients were allocated into the low-TG and high TG groups, using the cut-off value of 2648 mg/dL. The patients in the low-TG group had TG levels between 1000 and 2648 mg/dL, and the TG levels in the high-TG group were > 2648 mg/dL. The characteristics of the patients are summarized in Table 2. There was no significant difference in the sex ratio between the 2 groups (*P* = 0.52). Diabetes mellitus was present in 54.55% (36/66) of the patients in the low-TG group and in 60.26% (47/78) of the patients in the high-TG group (*P* = 0.602). Coronary artery disease was present in 7.58% (5/66) of the patients in the low-TG group and in 10.26% (8/78) of those in the high-TG group. The levels of bicarbonate (*P* = 0.002), and sodium (*P* = 0.003) were significantly higher in the low-TG group than those in the high-TG group. The levels of glucose (*P* = 0.022), total cholesterol (*P* = 0.002), and blood urea nitrogen (*P* = 0.037) were significantly higher in the high-TG group than in the low-TG group. There were no significant differences in the levels of amylase, lipase, total calcium, albumin, LDH, uric acid, and CRP.

**Table 2:** Characteristics of HTGP Patients with TG < 2648 and TG ≥ 2648.

	TG < 2648 (n = 66)	TG ≥ 2648(n = 78)	*χ*2 or *t*	*P* value ^a^
**Age, M±SE**	43.02±1.18	41.14±0.78	1.328	0.187
**Sex, n (%)**			0.415	0.520
Female	17(25.76)	25(32.05)		
Male	49(74.24)	53(67.95)		
**BMI, M±SE**	26.37±0.64	26.91±0.62	0.608	0.544
**DM (%)**			0.272	0.602
Yes	36(54.55)	47(60.26)		
**HTN (%)**			1.465	0.226
Yes	24(36.36)	20(25.64)		
**CAD (%)**			0.072	0.789
Yes	5(7.58)	8(10.26)		
**Pulmonary disease (%)**			0.058	0.849
Yes	7(10.6)	9(11.54)		
**CKD (%)**			0.226	0.637
Yes	6(9)	10(12.82)		
**Lipase (U/L)**	730.61±117.90	932.34±121.28	1.193	0.235
**Amylase (U/L)**	260.97±43.71	268.63±38.31	0.131	0.896
**Na (mmol/L)**	132.05±0.47	129.92±0.50	3.048	0.003*
**HCO3 (mEq/L)**	23.22±0.62	20.58±0.57	3.106	0.002*
**Glucose (mg/dl)**	198.58±16.68	248.81±14.11	2.315	0.022*
**Albumin (g/dL)**	3.68±0.06	3.73±0.07	0.574	0.567
**Total calcium (**mg/dL)	8.07±0.15	7.72±0.15	1.617	0.108
**Total cholesterol (**mg/dL)	294.44±14.61	393.61±27.85	3.153	0.002*
**LDH (**U/L)	318.44±36.59	371.40±32.71	1.081	0.282
**Uric acid (**mg/dL)	5.61±0.30	5.89±0.24	0.738	0.462
**CRP (mg/dL)**	8.86±1.76	9.10±1.70	0.098	0.922
**BUN (mg/dL)**	14.78±1.17	22.73±3.56	2.122	0.037*

BMI, body mass index; DM, diabetes mellitus; HTN, hypertension; CAD, coronary artery disease; CKD, chronic kidney disease; LDH, lactate dehydrogenase; CRP, C-reactive protein; BUN, blood urea nitrogen; M ± SE, Mean ± standard error

^a^Independent t-test or chi-square test.

* *P* < 0.05; Pulmonary disease was defined as a history of asthma or chronic obstructive pulmonary disease.

### Primary Endpoints

The primary endpoints, including organ failure, local and systemic complications, and HTGP severity, are summarized in Table 3. Local complications were evaluated by abdominal computed tomography scans. Local complications associated with ANC, APFC, or pseudocyst were present in 45.45% of the patients in the low-TG group and 69.23% of the patients in the high-TG group. Five patients (three with acute coronary syndrome and two with chronic obstructive pulmonary disease with acute exacerbation) in the high-TG group and one patient (with acute coronary syndrome) in the low-TG group had systemic complications. The moderately severe and severe forms of HTGP were found in 50% of the patients in the low-TG group and 74.36% of the patients in the high-TG group (*P* = 0.004). The method of severity classification was based on the revised Atlanta classification [25].

**Table 3:** Primary Endpoints versus TG Level in Patients with HTGP.

	TG < 2648 (n = 66)	TG ≥ 2648 (n = 78)	*χ*2 or *t*	*P* value ^a^
**Creatinine (mg/dl)**	0.98±0.09	1.51±0.24	2.039	0.044*
<1.9	59(89.39)	65(83.33)	0.650	0.420
≥1.9	7(10.61)	13(16.67)		
**Shock**			—	0.109 ^b^
Yes	2(3.03)	8(10.26)		
**Respiratory failure**			3.821	0.051
Yes	5(7.58)	16(20.51)		
**Organ failure numbers**			1.518	0.218
0	57(86.36)	60(76.92)		
≥1	9(13.64)	18(23.08)		
**Organ failure numbers**			—	0.008 ^b^^,^*
≤2	66(100.00)	70(89.74)		
≥3	0(0)	8(10.26)		
**Organ failure > 2 days**			3.144	0.076
Yes	5(7.58)	15(19.23)		
**Local complication on CT scan**			—	0.002 ^b^^,^*
ANC	6(9.09)	7(8.97)		
APFC	20(30.30)	46(58.97)		
Pseudocyst	4(6.06)	1(1.28)		
NIL	36(54.55)	24(30.77)		
**Systemic complication**			—	0.219 ^b^
Yes	1(1.52)	5(6.41)		
**Severity**			10.430	0.005*
Mild	33(50.00)	20(25.64)		
Moderately severe	28(42.42)	43(55.13)		
Severe	5(7.58)	15(19.23)		
**Severity**			8.103	0.004*
Mild	33(50.00)	20(25.64)		
Moderately + Severe	33(50.00)	58(74.36)		

ANC = acute necrotic collections, APFC = acute ​peripancreatic fluid collection.

M ± SE: Mean ± standard error

^a^Independent t-test or chi-square test

^b^Fisher’s exact test.

* *P* < 0.05

### Secondary Endpoints

The secondary endpoints included the ratio of ICU admission, hospitalization length, mortality, and recurrence of HTGP < 1 year after discharge (Table 4). The incidence of ICU admission, period of hospitalization, and mortality rates were higher in the patients in the high-TG group than in the low-TG group, but there was no statistical significance. All patients were followed-up for 1 year, and the recurrence rate was 24.62% in the low-TG group and 26.92% in the high-TG group (*P* = 0.903).

**Table 4:** Secondary Endpoints versus TG Level in Patients with HTGP.

	TG < 2648 (n = 66)	TG ≥ 2648 (n = 78)	*χ*2 or *t*	*P* value^a^
**ICU**			3.821	0.051
Yes	5(7.58)	16(20.51)		
**Hospital day**	8.35±1.01	11.12±1.32	1.659	0.100
**Mortality**			—	0.070 ^b^
Yes	1(1.52)	7(8.97)		
**Recurrence in one year**			0.015	0.903
Yes	16(24.62)	21(26.92)		

ICU, intensive care unit; M ± SE: Mean ± standard error

^a^Independent t-test or chi-square test

^b^Fisher’s exact test

## Discussion

This study retrospectively compared the clinical characteristics, initial laboratory data, primary endpoints, and secondary endpoints of the low-TG and high-TG groups. The high-TG group had significantly higher levels of glucose, total cholesterol, and blood urea nitrogen, and lower levels of sodium and bicarbonate than the low-TG group. Analysis of the primary endpoints showed that the high-TG group had a significantly higher incidence of local complications and more severe form of HTGP than the low-TG group. Analysis of the secondary endpoints showed that there were longer hospitalization days and higher mortality and ICU admission rates in the high-TG group than in the low-TG group, although there were no significant differences.

Serum with hyperlipidemia could artifactually cause pseudohyponatremia. Hyperlipidemia increases the portion of nonaqueous components in the serum, and the decrease in the water percentage of the plasma causes less sodium in a given volume aliquot, although the concentration of sodium in the water phase is the same [26,27]. In our study, the lower sodium level in the high-TG group may be related to a higher percentage of the nonaqueous component in serum. The lower level of bicarbonate in the high-TG group may be related to the higher rates of respiratory failure, shock, or impaired renal function in the patients. Plasma glucose was verified as one factor in causing acute stress [28]. Higher plasma glucose level in the high-TG group may be a physiological response to a more severe form of pancreatitis in these patients, in addition to a higher incidence of diabetes mellitus.

Many scoring systems, including Ranson's criteria, the Acute Physiology and Chronic Health Examination (APACHE) II score, and Bedside Index of Severity in Acute Pancreatitis (BISAP) score, are used to evaluate the prognosis of AP, but none have been proven to be accurate in assessing severity [29–30]. Persistent organ failure is widely accepted as a reliable predictor of adverse outcomes in AP [31–33]. The revised Atlanta classification system defines organ failure involving respiratory, renal, or cardiovascular systems > 48 h as severe AP, and the presence of transient organ failure (< 48h), local complication or systemic complication as moderately severe AP [25]. To our knowledge, we are the first to apply this more precise classification system to assess severity of HTGP and to further observe and show that higher TG level may be related to a more severe form (moderately severe + severe) of HTGP. Our study showed that 63.1% (91/144) of all patients with HTGP had the more severe form, which also corroborated the previous studies that showed that the severe form could account for 56–71% of all patients with HTGP, even with different classification methods [16, 21]. A total of 8 deaths occurred during our study, and all cases of death had at least two organs failures. Furthermore, 8 patients had 3 organs failures, and 7 of them died. This implies multiple organ failure on initial admission could indicate a poor prognosis; thus, organ failure has a major role in the revised Atlanta classification system for AP. Our study also showed significant higher incidence of local complications on CT scan in the high-TG group, especially APFC. Computed tomography severity index is a widely used method to assess the severity of AP, and APFC could be classified as grade D or E using this classification [34]. Therefore, the AP severity in the high-TG group is more severe than that in the low-TG group based on the CT severity index.

The severity of HTGP is dependent on both the lipotoxicity from free fatty acids and a series of inflammatory injuries caused by pancreatitis itself. Positive correlation between the lipotoxicity effect and decrease in the plasma calcium level has been previously observed [17, 35]. In our study, patients in the high-TG group had a lower level of total calcium, which may imply that more lipotoxicity occurs in patients with HTGP with a higher TG level and is one of the factors leading to poor prognosis. However, the relationship between lipotoxicity and TG level requires further studies for elucidation.

Our study had several limitations. First, because this study was retrospective, unintended bias may have occurred. Second, the etiology of HTG could not be identified; therefore, subgroup analysis could not be performed. Third, most patients with impaired renal function underwent non-contrast abdominal computed tomography imaging. It is difficult to differentiate necrosis or cyst lesions by using non-contrast computed tomography, which may lead to difficulty in diagnosis of local complication. Fourth, the number of cases was small.

## Conclusions

We retrospectively reviewed the records of 144 patients with HTGP and allocated them to low-TG and high-TG groups based on the cut-off value of 2648 mg/dL, which was derived from the receiver operating characteristic (ROC) curve between TG level and severity of HTGP. A more severe form of pancreatitis was observed in the high-TG group than in the low-TG group, which implies that higher TG level may be associated with poor prognosis in patients with HTGP. Further large randomized trials are needed to evaluate the effect and mechanism of TG level on HTGP.

## Supporting Information

S1 File Institutional Review Board. (PDF)

S1 Table Modified Marshall Scoring System for Organ Dysfunction. (DOC)

S2 Table Characteristics of HTGP Patients with TG < 2648 and TG ≧ 2648. (DOC)

S3 Table Primary Endpoints versus TG Level in Patients with HTGP. (DOC)

S4 Table Secondary Endpoints versus TG Level in Patients with HTGP. (DOC)
