# Supplementary material for: Relationship between Plasma Triglyceride Level and Severity of Hypertriglyceridemic Pancreatitis
Source: PLoS One. 2016 Oct 11;11(10):e0163984. doi: 10.1371/journal.pone.0163984 (PMC5058492; doi:10.1371/journal.pone.0163984)
Supplement: S1 File — (PDF) [file pone.0163984.s001.pdf]

TEL: 886-2-87923311 ext 10552
FAX:886-2-2793-6049 / 2792-9201
E-mail: tsghirb@ndmctsgh.edu.tw

國防醫學院三軍總醫院
人體試驗審議會

11490 台北市內湖區成功路二段
325 號 醫療大樓五樓 5113 室
No. 325, Sec.2, Cheng-Kung Rd.
Neihu 11490, Taipei, Taiwan, R.O.C

人體試驗計畫同意函

本審議會核准編號：1-105-05-041

計畫名稱：探討血液中三酸甘油脂值與高三酸甘油脂引發的胰臟炎之關係

執行機構：三軍總醫院

計畫主持人：內科部王勝輝醫師

計畫書版本日期：V 2.0_20160329

個案報告表版本日期：V1.0_20160320

本會審核通過之其他文件版本及日期：中文摘要 V2.0_20160329

業經本院 2016 年 4 月 17 日人體試驗審議會第一審議會審查通過，該計畫案經評估屬低度風險，(持續審查頻率為每年一次)，有效期限至 2017 年 4 月 16 日，特此證明。本審議會的運作，遵循藥品優良臨床試驗準則及政府相關法律規章。計畫主持人應於同意函有效期屆滿前二個月，提出展延申請，本案須經本院人體試驗審議會通過後，方可繼續執行。

Letter of Approval
Institutional Review Board, Tri-Service General Hospital

TSGHIRB No.: 1-105-05-041

Protocol title: The relation between plasma triglyceride level and hypertriglyceridemia-induced acute pancreatitis.

Research institution: Tri-Service General Hospital

Principle investigator: Dr. Sheng-Huei Wang

Protocol version: V 2.0_20160329

Case Report Form: V1.0_20160320

Other documents: Chinese Abstract V1.0_20160320

on 04/17/2016, the Institutional Review Board I of the Tri-Service General Hospital approved the above-named application. The board is organized and operated in compliance with International Conference on Harmonization (ICH) / WHO Good Clinical Practice (GCP) and applicable laws and regulations.

This approval is valid for 1 year till 04/16/2017. The principle investigator is required to submit the application for extension 2 months before the expiration date.

三軍總醫院 TSGHIRB 人體試驗審議會

Institutional Review Board

余慕賢 Yu Mu Hsien

Chairman__________________________.
