# Supplementary material for: Relationship between Plasma Triglyceride Level and Severity of Hypertriglyceridemic Pancreatitis
Source: PLoS One. 2016 Oct 11;11(10):e0163984. doi: 10.1371/journal.pone.0163984 (PMC5058492; doi:10.1371/journal.pone.0163984)
Supplement: S1 Table — (DOC) [file pone.0163984.s002.doc]

Table 1. Modified Marshall Scoring System for Organ Dysfunction.

| **Organ system** | **Score: 0** | **1** | **2** | **3** | **4** |
| --- | --- | --- | --- | --- | --- |
| **Respiratory (PaO_2_/FiO_2_)** | >400 | 301–400 | 201–300 | 101–200 | ≤101 |
| **Renal (serum creatinine, mg/dl)** | <1.4 | 1.4–1.8 | 1.9–3.6 | 3.6–4.9 | >4.9 |
| **Cardiovascular (systolic blood pressure, mm Hg)** # | >90 | <90, fluid response | <90, no fluid response | <90, pH <7.3 | <90, pH <7.2 |

A score of ≥2 in any system defines the presence of organ failure.

Organ failure resolving ≤48 h is defined as transient organ failure; organ failure persisting >48 h is defined as persistent organ failure

# without inotropic agents

Reference: 25
