# Supplementary material for: Relationship between Plasma Triglyceride Level and Severity of Hypertriglyceridemic Pancreatitis
Source: PLoS One. 2016 Oct 11;11(10):e0163984. doi: 10.1371/journal.pone.0163984 (PMC5058492; doi:10.1371/journal.pone.0163984)
Supplement: S2 Table — (DOC) [file pone.0163984.s003.doc]

**Table 2. Characteristics of HTGP Patients with TG < 2648 and TG ≥ 2648.**

|  | **TG < 2648 (n = 66)** | **TG ≥ 2648(n = 78)** | ***χ*2 or *t*** | ***P* value** **^a^** |
| --- | --- | --- | --- | --- |
| **Age, M±SE** | 43.02±1.18 | 41.14±0.78 | 1.328 | 0.187 |
| **Sex, n (%)** |  |  | 0.415 | 0.520 |
| Female | 17(25.76) | 25(32.05) |  |  |
| Male | 49(74.24) | 53(67.95) |  |  |
| **BMI, M±SE** | 26.37±0.64 | 26.91±0.62 | 0.608 | 0.544 |
| **DM (%)** |  |  | 0.272 | 0.602 |
| Yes | 36(54.55) | 47(60.26) |  |  |
| **HTN (%)** |  |  | 1.465 | 0.226 |
| Yes | 24(36.36) | 20(25.64) |  |  |
| **CAD (%)** |  |  | 0.072 | 0.789 |
| Yes | 5(7.58) | 8(10.26) |  |  |
| **Pulmonary disease (%)** |  |  | 0.058 | 0.849 |
| Yes | 7(10.6) | 9(11.54) |  |  |
| **CKD (%)** |  |  | 0.226 | 0.637 |
| Yes | 6(9) | 10(12.82) |  |  |
| **Lipase (U/L)** | 730.61±117.90 | 932.34±121.28 | 1.193 | 0.235 |
| **Amylase (U/L)** | 260.97±43.71 | 268.63±38.31 | 0.131 | 0.896 |
| **Na (mmol/L)** | 132.05±0.47 | 129.92±0.50 | 3.048 | 0.003^*^ |
| **HCO3 (mEq/L)** | 23.22±0.62 | 20.58±0.57 | 3.106 | 0.002^*^ |
| **Glucose (mg/dl)** | 198.58±16.68 | 248.81±14.11 | 2.315 | 0.022^*^ |
| **Albumin (g/dL)** | 3.68±0.06 | 3.73±0.07 | 0.574 | 0.567 |
| **Total calcium (**mg/dL**)** | 8.07±0.15 | 7.72±0.15 | 1.617 | 0.108 |
| **Total cholesterol (**mg/dL**)** | 294.44±14.61 | 393.61±27.85 | 3.153 | 0.002^*^ |
| **LDH (**U/L**)** | 318.44±36.59 | 371.40±32.71 | 1.081 | 0.282 |
| **Uric acid (**mg/dL**)** | 5.61±0.30 | 5.89±0.24 | 0.738 | 0.462 |
| **CRP (mg/dL)** | 8.86±1.76 | 9.10±1.70 | 0.098 | 0.922 |
| **BUN (mg/dL)** | 14.78±1.17 | 22.73±3.56 | 2.122 | 0.037^*^ |

BMI, body mass index; DM, diabetes mellitus; HTN, hypertension; CAD, coronary artery disease; CKD, chronic kidney disease; LDH, lactate dehydrogenase; CRP, C-reactive protein; BUN, blood urea nitrogen; M ± SE, Mean ± standard error; ^a^Independent t-test or chi-square test. * *P* < 0.05; Pulmonary disease was defined as a history of asthma or chronic obstructive pulmonary disease.
