# Supplementary material for: Relationship between Plasma Triglyceride Level and Severity of Hypertriglyceridemic Pancreatitis
Source: PLoS One. 2016 Oct 11;11(10):e0163984. doi: 10.1371/journal.pone.0163984 (PMC5058492; doi:10.1371/journal.pone.0163984)
Supplement: S3 Table — (DOC) [file pone.0163984.s004.doc]

**Table 3. Primary Endpoints versus TG Level in Patients with HTGP.**

|  | **TG ≥ 2648 (n = 78)** | **TG ≥ 2648 (n = 78)** | ***χ*2 or *t*** | ***P* value** **^a^** |
| --- | --- | --- | --- | --- |
| **Creatinine (mg/dl)** | 0.98±0.09 | 1.51±0.24 | 2.039 | 0.044* |
| <1.9 | 59(89.39) | 65(83.33) | 0.650 | 0.420 |
| ≥1.9 | 7(10.61) | 13(16.67) |  |  |
| **Shock** |  |  | -- | 0.109 ^b^ |
| Yes | 2(3.03) | 8(10.26) |  |  |
| **Respiratory failure** |  |  | 3.821 | 0.051 |
| Yes | 5(7.58) | 16(20.51) |  |  |
| **Organ failure numbers** |  |  | 1.518 | 0.218 |
| 0 | 57(86.36) | 60(76.92) |  |  |
| ≥1 | 9(13.64) | 18(23.08) |  |  |
| **Organ failure numbers** |  |  | -- | 0.008 ^b^,* |
| ≤2 | 66(100.00) | 70(89.74) |  |  |
| ≥3 | 0(0) | 8(10.26) |  |  |
| **Organ failure > 2 days** |  |  | 3.144 | 0.076 |
| Yes | 5(7.58) | 15(19.23) |  |  |
| **Local complication on CT scan** |  |  | -- | 0.002 ^b^,* |
| ANC | 6(9.09) | 7(8.97) |  |  |
| APFC | 20(30.30) | 46(58.97) |  |  |
| Pseudocyst | 4(6.06) | 1(1.28) |  |  |
| NIL | 36(54.55) | 24(30.77) |  |  |
| **Systemic complication** |  |  | -- | 0.219 ^b^ |
| Yes | 1(1.52) | 5(6.41) |  |  |
| **Severity** |  |  | 10.430 | 0.005* |
| Mild | 33(50.00) | 20(25.64) |  |  |
| Moderately severe | 28(42.42) | 43(55.13) |  |  |
| Severe | 5(7.58) | 15(19.23) |  |  |
| **Severity** |  |  | 8.103 | 0.004* |
| Mild | 33(50.00) | 20(25.64) |  |  |
| Moderately + Severe | 33(50.00) | 58(74.36) |  |  |

ANC = acute necrotic collections, APFC = acute ​peripancreatic fluid collection.

M ± SE: Mean ± standard error, ^a^Independent t-test or chi-square test, ^b^Fisher’s exact test.

* *P* < 0.05
