# Supplementary material for: Relationship between Plasma Triglyceride Level and Severity of Hypertriglyceridemic Pancreatitis
Source: PLoS One. 2016 Oct 11;11(10):e0163984. doi: 10.1371/journal.pone.0163984 (PMC5058492; doi:10.1371/journal.pone.0163984)
Supplement: S4 Table — (DOC) [file pone.0163984.s005.doc]

**Table 4.** **Secondary Endpoints versus TG Level in Patients with HTGP.**

|  | **TG < 2648 (n = 66)** | **TG ≥ 2648 (n = 78)** | ***χ*2 or *t*** | ***P* value^a^** |
| --- | --- | --- | --- | --- |
| **ICU** |  |  | 3.821 | 0.051 |
| Yes | 5(7.58) | 16(20.51) |  |  |
| **Hospital day** | 8.35±1.01 | 11.12±1.32 | 1.659 | 0.100 |
| **Mortality** |  |  | -- | 0.070 ^b^ |
| Yes | 1(1.52) | 7(8.97) |  |  |
| **Recurrence in one year** |  |  | 0.015 | 0.903 |
| Yes | 16(24.62) | 21(26.92) |  |  |

ICU, intensive care unit; M ± SE: Mean ± standard error,

^a^Independent t-test or chi-square test; ^b^Fisher’s exact test
